# Diagnosing capillary leak in critically ill patients: development of an innovative scoring instrument for non-invasive detection

**DOI:** 10.1186/s13613-021-00965-8

**Published:** 2021-12-15

**Authors:** Jakob Wollborn, Lars O. Hassenzahl, Daniel Reker, Hans Felix Staehle, Anne Marie Omlor, Wolfgang Baar, Kai B. Kaufmann, Felix Ulbrich, Christian Wunder, Stefan Utzolino, Hartmut Buerkle, Johannes Kalbhenn, Sebastian Heinrich, Ulrich Goebel

**Affiliations:** 1 Department of Anesthesiology and Critical Care, Medical Center, University of Freiburg, Freiburg im Breisgau, Germany; 2 Department of Anesthesiology, Perioperative and Pain Medicine, Brigham and Women’s Hospital, Harvard Medical School, 75 Francis Street, Boston, MA 02115 USA; 3 Department of Biomedical Engineering, Duke University, Durham, NC USA; 4 Department of Anesthesiology and Critical Care, Robert-Bosch-Krankenhaus, Stuttgart, Germany; 5 Department of General and Visceral Surgery, Medical Center, University of Freiburg, Freiburg im Breisgau, Germany; 6 Faculty of Medicine, University of Freiburg, Freiburg im Breisgau, Germany; 7 Department of Anesthesiology and Critical Care, St. Franziskus-Hospital, Muenster, Germany

**Keywords:** Capillary leak syndrome, Critical care, Sepsis, Fluid balance, Endothelial permeability

## Abstract

**Background:**

The concomitant occurrence of the symptoms intravascular hypovolemia, peripheral edema and hemodynamic instability is typically named Capillary Leak Syndrome (CLS) and often occurs in surgical critical ill patients. However, neither a unitary definition nor standardized diagnostic criteria exist so far. We aimed to investigate common characteristics of this phenomenon with a subsequent scoring system, determining whether CLS contributes to mortality.

**Methods:**

We conducted this single-center, observational, multidisciplinary, prospective trial in two separately run surgical ICUs of a tertiary academic medical center. 200 surgical patients admitted to the ICU and 30 healthy volunteers were included. Patients were clinically diagnosed as CLS or No-CLS group (each N = 100) according to the grade of edema, intravascular hypovolemia, hemodynamic instability, and positive fluid balance by two independent attending physicians with > 10 years of experience in ICU. We performed daily measurements with non-invasive body impedance electrical analysis, ultrasound and analysis of serum biomarkers to generate objective diagnostic criteria. Receiver operating characteristics were used, while we developed machine learning models to increase diagnostic specifications for our scoring model.

**Results:**

The 30-day mortility was increased among CLS patients (12 vs. 1%, *P* = 0.002), while showing higher SOFA-scores. Extracellular water was increased in patients with CLS with higher echogenicity of subcutaneous tissue [29(24–31) vs. 19(16–21), *P* < 0.001]. Biomarkers showed characteristic alterations, especially with an increased angiopoietin-2 concentration in CLS [9.9(6.2–17.3) vs. 3.7(2.6–5.6)ng/mL, *P* < 0.001]. We developed a score using seven parameters (echogenicity, SOFA-score, angiopoietin-2, syndecan-1, ICAM-1, lactate and interleukin-6). A Random Forest prediction model boosted its diagnostic characteristics (AUC 0.963, *P* < 0.001), while a two-parameter decision tree model showed good specifications (AUC 0.865).

**Conclusions:**

Diagnosis of CLS in critically ill patients is feasible by objective, non-invasive parameters using the *CLS-Score*. A simplified two-parameter diagnostic approach can enhance clinical utility. CLS contributes to mortality and should, therefore, classified as an independent entity.

*Trial Registration*: German Clinical Trials Registry (DRKS No. 00012713), Date of registration 10/05/2017, www.drks.de

**Graphical Abstract:**

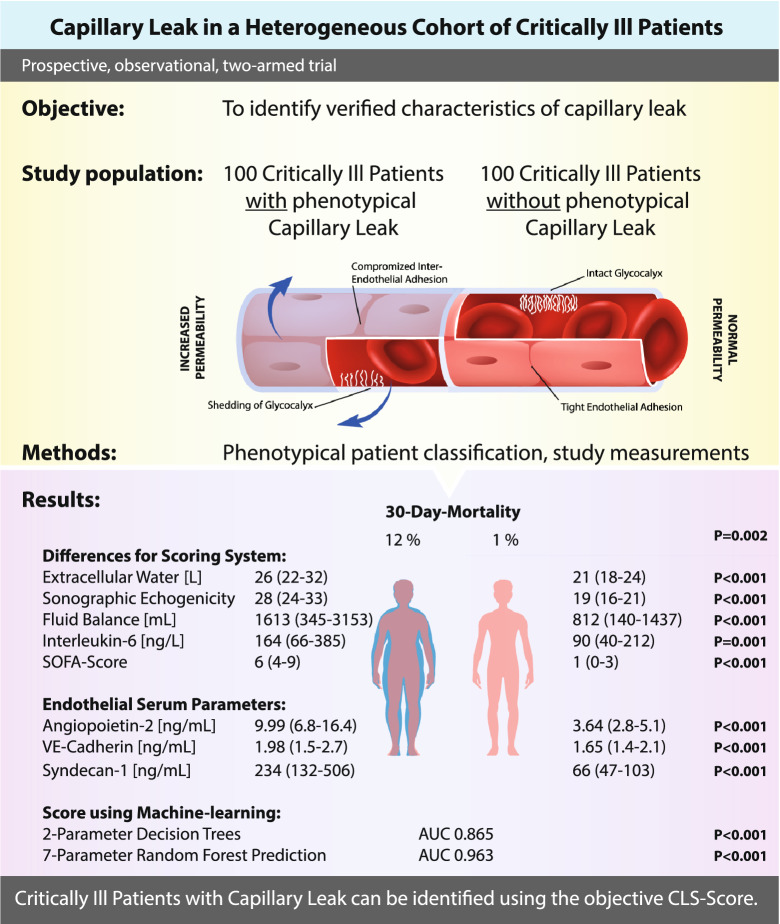

**Supplementary Information:**

The online version contains supplementary material available at 10.1186/s13613-021-00965-8.

## Background

Critically ill patients regularly present with the concomitant symptoms of intravascular hypovolemia needing fluid resuscitation and leading to positive fluid balance, considerable edema and hemodynamic instability. This phenotype is recognized in clinical routine and described as capillary leak syndrome (CLS). To this day, however, no consensus definition for CLS is available. This has not changed despite previous attempts to evaluate determinants of CLS in critically ill patients [1]. Although various studies describe mechanisms to modulate endothelial permeability [2–5], neither diagnostic criteria nor uniformed clinical terminology is available. It remains unclear whether CLS merely represents an epiphenomenon of critical illness or if it needs to be regarded as an independent pathogenic entity. Of note, the use of the term CLS in our study is not synonymous with “Clarkson’s disease” (sometimes also referred as “idiopathic systemic capillary leak syndrome”) which describes a rare, but severe pathology of unknown etiology. Patients with a history of Clarkson’s syndrome were excluded from our study. The use of the term CLS in our manuscript goes in line with previous work in the intensive care literature [1, 6].

CLS in the ICU may not be limited to sepsis [7], but can also be seen in patients after cardiopulmonary bypass [8–10], anaphylaxis [11], and thermal injury [12]. Investigations into its pathophysiology have revealed mechanisms related to CLS: while a pro-inflammatory state increases vascular endothelial permeability [13], integrity of inter-cellular junctions of endothelial cells become compromised [4] and glycocalyx is shedded [14–17]. For prevention and treatment of CLS, experimental pharmacological approaches to attenuate these mechanisms showed promising results in pre-clinical models [18–20]. It has been hypothesized that novel strategies for adjunctive sepsis therapy should focus on endothelial permeability [14, 21].

Despite common perception of its high relevance [22], the authors are not aware of any clinical study assessing the effect of CLS on organ dysfunction and mortality. This may be due to the fact that no diagnostic criteria exist. However, its concomitant circumstances such as a proinflammatory state as well as a positive fluid balance have been linked to increased mortality in the ICU: Concentrations of serum cytokines tend to be higher in non-survivors of critical illness [23], while a positive fluid balance reflects an independent prognostic factor in patients with sepsis [24].

We hypothesized that CLS patients present with distinct characteristics and differ from patients without CLS. It was the aim of this study to identify patterns and establish a scoring system with prognostic strength.

## Methods

### Design and setting

The trial was designed in a prospective, multidisciplinary, observational approach. Reporting complied with the TRIPOD (Transparent reporting of a multivariable prediction model for individual prognosis or diagnosis) statement [25]. Surgical patients were recruited from two intensive care units at the Medical Center of the University of Freiburg, Germany from October 2017 to July 2019. The study was approved by the institutional review board (Freiburg, EK-Nr. 68/17) and was registered (DRKS No. 00012713). All healthy volunteers, patients, their respective next-of-kin or legal guardian needed to provide consent for study inclusion. Patients were clinically classified as CLS or No-CLS according to expert evaluation (two independent consultant physicians with > 10 years of experience in intensive care medicine according to the following clinical criteria: edema, intravascular hypovolemia, positive fluid balance and hemodynamic instability). The experts were blinded, were not allowed to access data other than aforementioned parameters, not involved in the patient care, and not aware of patient outcomes. Two hundred and twenty patients were screened for study inclusion. Thirty healthy volunteers were, furthermore, included to determine reference values for study measurements.

### Study population

We assessed the eligibility of patients admitted to our intensive care units. They were categorized as CLS and No-CLS patients based on the early presentation in the ICU by clinical criteria (N = 100 per group). Inclusion criteria were defined as age ≥ 18 years, estimated length of stay in the ICU ≥ 48 h and informed consent. Patients were excluded if one or more of the following criteria applied: Refusal to participate, infection with HIV, viral hepatitis, idiopathic capillary leak syndrome (“Clarkson’s disease”), hereditary C1-esterase deficiency, recurrent angioedema, pre-existing chronic kidney failure requiring dialysis, and pre-existing hepatic impairment with a MELD score ≥ 20.

### Patient care

Daily evaluation and measurements were performed after morning rounds in the respective ICU. Fluid therapy was guided by a clinical examination, a positive fluid challenge or passive leg raise test with an increase in blood pressure or cardiac index, respectively. Available physiologic variables were taken into consideration as well as other noninvasive or invasive monitoring, as available. Only balanced crystalloid solutions were used for fluid resuscitation in our patients.

### Data collection

For an in-depth evaluation of fluid homeostasis, bioelectrical impedance (BIA) measurements were performed daily. For BIA, the Nutriguard-MS system was used (Data Input GmbH, Poecking, Germany) [26–28]. Measurement was conducted in a hand-to-foot approach with the patient strictly supine and free from measurement confounders. Data analyses was performed using the customized NutriPlus^©^ software (Data Input GmbH, Poecking, Germany). Ultrasound was used to quantify peripheral and pulmonary edema. Using standardized views, echo free space was evaluated by measuring the tissue-free, subcutaneous distance [29]. Routinely available parameters such as demographics, medication, and laboratory values were collected from the patients’ electronic charts. Patients’ serum was collected to perform subsequent ELISA and FACS analyses (see Additional File 1).

### Statistical analysis

Analyses were performed with SPSS® Statistics (V26, Chicago, USA) and GraphPad Prism (V8, San Diego, USA). *P* values ≤ 0.05 were considered statistically significant. For variables without a consensus-based reference range (e.g., body impedance electrical analysis, ultrasound parameters, and endothelial biomarkers), data from healthy volunteers were collected, analyzed and the 5–95% percentile was expressed as the reference for our study. The study data was then analyzed in a univariate approach: if continuous data showed normal distribution, Student’s *t* test was used. For non-normally distributed variables, we used Mann–Whitney *U* test. Categorial data was analyzed with *X*^2^ test. Continuous variables were dichotomized according to the 75% percentile. Only the variables showing statistically significant differences were included in further analyses. A Kaplan–Meier curve was created for survival analysis and the log-rank score was calculated. A stepwise multivariate binary logistic regression model was built to determine odds ratios and 95% confidence intervals. A forward selection was followed by a backward conditional approach for confirmation of results. Consecutively, a linear scoring system was built from the parameters showing promising predictive values: SOFA-Score, echogenicity of subcutaneous tissue, serum-lactate, angiopoietin-2, syndecan-1, IL-6, and ICAM-1. For the seven parameter scoring system, one point was each given for values above the 75^th^ percentile of the whole patient cohort with a score cutoff of  ≥  2 (out of 7 possible points). Receiver Operating Characteristic (ROC) analysis was performed and the area under the curve (AUC) was calculated. The score was then re-evaluated within our data set.

For data mining and machine learning, KNIME (V4.1.2, Zurich, Switzerland) was used. If any of the patients missed an attribute necessary for model fitting, this patient was removed from the analysis. Models were trained with standard parameters and were evaluated in 10 × tenfold cross validations. For every training fold, data was standardized (Z-score normalized). Standardization of the test set was performed separately according to parameters of the training data to avoid carrying information from the test data into the training data. This training data was then fed to the machine learning algorithms with default model parameters. Specifically, we used a Decision Tree (Gini index, no pruning, min. 2 records per node, average split point), a Support-Vector Machine (polynomial kernel with bias, gamma, and power of 1.0), a Deep Neural Network (Multilayer Feedforward Network with RProp training, 3 hidden layers with 10 neurons per layer), a Random Forest (100 trees, Information Gain Ratio), Gradient Boosted Trees (100 trees, learning rate 0.1, maximum tree depth 4), a Probabilistic Neural Network (following the Dynamic Decay Adjustment, theta minus 0.2, theta plus 0.4) and a Naïve Bayes Learner (default probability and minimum standard deviation 0.0001, threshold 0.0). For all models, predictive confidence was extracted to enable ROC AUC calculation and their statistical significance was analyzed following a Wilcoxon–Mann–Whitney test. Individual AUC values were acquired and we report mean values, standard deviation, and maximum *P *value as aggregate statistics for the ten separate analyses. As an additional test of robustness, we reimplemented the most predictive Random Forest model in Google Colab using the scikit-learn library and confirmed a similar ROC AUC values of 0.96 in tenfold cross validation. For predictors, we selected all available classification models available through the KNIME data analytics software (V4.0.2). To assess the diagnostic specifications of our models, the six different machine-learning algorithms were evaluated regarding its predictive capabilities for the promising 7-parameter data set. This evaluation used tenfold cross validations to enable the contextualization of model performance on data that was not used for model fitting.

## Results:

**Fig. 1:**
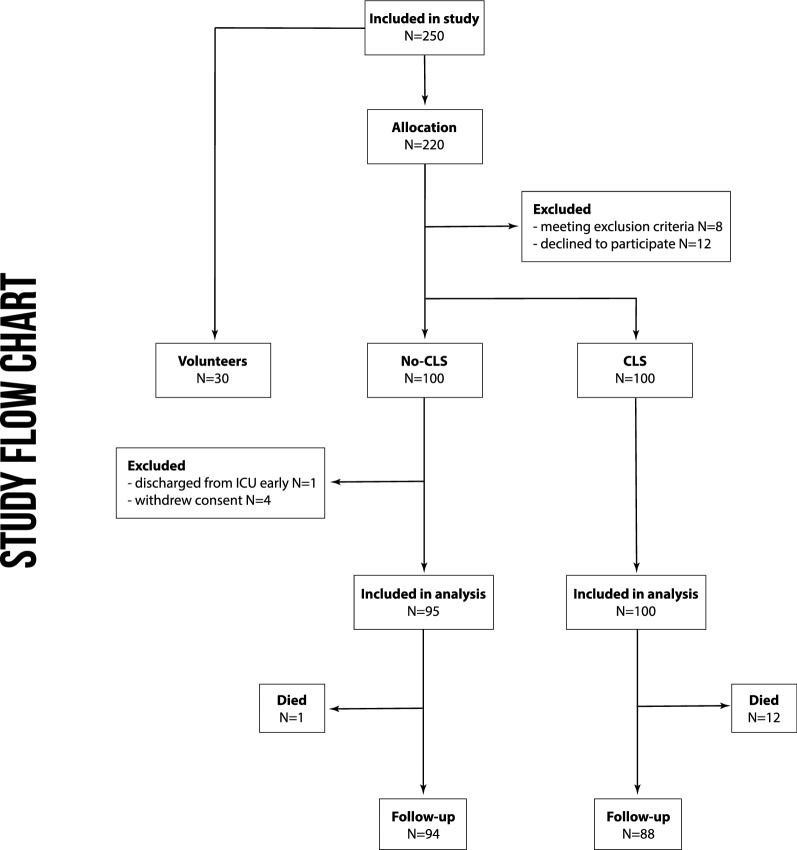
Study flow chart

250 individuals were assessed for eligibility (see Fig. 1). 30 healthy volunteers were included to define a reference range of study parameters. 12 patients declined to participate and 8 patients met exclusion criteria. 100 patients were recruited each in the CLS and in the No-CLS group. Within the No-CLS group, four patients withdrew consent and one patient was discharged early, leaving 95 and 100 patients, respectively, to be included in the analysis.

Assessing 30-day mortality, twelve patients died in the CLS group compared to one patient in the No-CLS group (12% vs. 1%, log-rank *P* = 0.002, see Additional File 2). The majority of patients died from septic shock or ARDS (see Additional File 3).

Our group of healthy volunteers, which was needed to reference study measurements showed 50% males and a median age of 22 [22–24] (see Additional File 4).

Patient characteristics (see Table 1) revealed that patients presenting with CLS were older (67 ± 13 vs. 62 ± 17, *P* = 0.021) and showed a higher body-mass index prior to diagnosis of CLS (28 ± 6 vs. 26 ± 5, *P* = 0.044). In our cohort of CLS patients, chronic obstructive pulmonary disease was significantly more frequent compared to the No-CLS group (12 vs. 3%, *P* = 0.031). Prior medication included the use of ß-Receptor-Blockers in 47% in the CLS group compared to 22% among No-CLS patients (*P* = 0.001). Diuretics were used among 27% of CLS and 12% of No-CLS patients (*P* = 0.011). In the CLS group, patients were more frequently diagnosed with sepsis (56 vs. 5%, *P* < 0.001), acute respiratory distress syndrome (6 vs. 0%, *P* = 0.03), acute kidney injury (58 vs. 32%, *P* = 0.003), and shock of any cause (41 vs. 11%, *P* < 0.001). Among the patients undergoing surgery prior to admission, blood loss was higher in the patients classified as CLS (650 [139–2050] vs. 300 [175–525] mL, *P* = 0.003) and fluid balance was increased (4700 [3500–8300] vs. 3250 [2150–5338] mL, *P* = 0.002). The No-CLS group included more patients with pancreatic surgery (13 vs. 1%, *P* = 0.001), while emergency laparotomy (17 vs. 1%, *P* < 0.001) and vascular surgery (6 vs. 0%, *P* = 0.015) was more often in the CLS group.

**Table 1:** Patient characteristics in the No-CLS and CLS groups

	No-CLS (*N* = 95)	CLS (*N* = 100)	*P* value
General patient characteristics			
Age (mean ± SD)	62 ± 17	67 ± 13	**0.021**
BMI (mean ± SD)	26 ± 5	28 ± 6	**0.044**
Male (%)	55 (58%)	58 (58%)	0.749
Died on ICU (%)	1 (1%)	12 (12%)	**0.003**
Past medical history			
Hypertension (%)	48 (51%)	54 (54%)	0.774
Myocardial infarction (%)	4 (4%)	6 (6%)	0.750
Chronic obstructive pulmonary disease (%)	3 (3%)	12 (12%)	**0.031**
Chronic heart failure (%)	0 (0%)	1 (1%)	1
Atrial fibrillation (%)	10 (11%)	20 (20%)	0.112
Prior medication			
ACE inhibitors (%)	25 (26%)	29 (29%)	0.874
ß-Receptor-Blockers (%)	21 (22%)	47 (47%)	**0.001**
Calcium channel blockers (%)	6 (6%)	5 (5%)	0.760
Diuretics (%)	11 (12%)	27 (27%)	**0.011**
Statins (%)	13 (14%)	17 (17%)	0.693
Aspirin (%)	17 (18%)	23 (23%)	0.484
NSAIDs (%)	6 (6%)	5 (5%)	0.760
Antidiabetics (%)	12 (13%)	10 (10%)	0.508
Antiasthmatics (%)	1 (1%)	0 (0%)	0.469
Psychopharmaceuticals (%)	3 (3%)	12 (12%)	**0.031**
Diagnosis on ICU			
Sepsis (%)	5 (5%)	56 (56%)	** < 0.001**
ARDS (%)	0 (0%)	6 (6%)	**0.030**
AKI (%)	32 (34%)	58 (58%)	**0.003**
ALF (%)	0 (0%)	1 (1%)	1
Shock (%)	10 (11%)	41 (41%)	** < 0.001**
Characteristics during surgery			
Blood loss, mL (median ± IQR)	300 (175–525)	650 (139–2050)	**0.003**
Fluid balance, mL (median ± IQR)	3250 (2150–5338)	4700 (3500–8300)	**0.002**
Gastric surgery	3 (3%)	0	0.073
Pancreatic surgery	12 (13%)	1 (1%)	**0.001**
Hepatobiliary surgery	9 (10%)	2 (2%)	0.05
Bowel resection	22 (23%)	31 (31%)	0.219
Tumor Debulking	12 (13%)	9 (9%)	0.414
Cystectomy	3 (3%)	0	0.073
Thoracoabdominal surgery	7 (7%)	4 (4%)	0.199
Emergency Laparotomy	1 (1%)	17 (17%)	** < 0.001**
Vascular surgery	0	6 (6%)	**0.015**
Gynecological surgery	8 (8%)	4 (4%)	0.199
Other	18 (19%)	26 (26%)	0.239

Bold numbers reflect statistical significance (*P* ≤ 0.05)

*BMI* body mass index, *NSAID* non-steroidal anti-inflammatory drugs, *ARDS* acute respiratory distress syndrome, *AKI* acute kidney injury, *ALF* acute liver failure

**Fig. 2:**
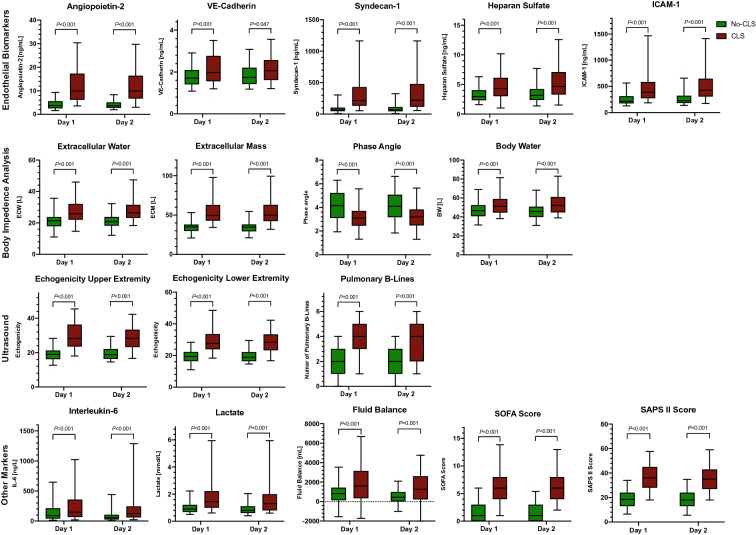
Study parameters in the No-CLS and CLS groups on ICU days 1 and 2 (Box plots with 5–95% whiskers)

Our study measurements can be summarized as follows (see Fig. 2 and Additional File 5): CLS patients required more catecholamine support than No-CLS patients (day 1: 95 vs. 85%, *P* = 0.022), had a higher fluid balance (day 1: 1613[345–3153] vs. 812[140–1437] mL, *P* < 0.001), increased need for fluid input (day 1: 3280[2226–4854] vs. 2077[1421–2945] mL, *P* < 0.001), higher lactate levels (day 1: 1.5[1–2.2] vs. 0.9[0.7–1.2] mmol/L, *P* < 0.001), and a lower hemoglobin concentration (day 1: 8.6[8–9.8] vs. 10.6[8.5–12.1] g/dL, *P* < 0.001). Disease severity scores showed a higher SAPS II (day 1: 36 [28–45] vs. 19[13–24], *P* < 0.001), SOFA (day 1: 6[4–9] vs. 1[0–3] mL, *P* < 0.001), and APACHE II score (day 1: 12[9–16] vs. 6[4–9] mL, *P* < 0.001).

Body impedance electrical analysis revealed increased extracellular water (day 1: 26[22–32] vs. 21[18–24] L, *P* < 0.001) and body water (day 1: 51[45–59] vs. 47[41–52] L, *P* = 0.001) among CLS patients, whereas the phase angle was lower (day 1: 2.6[2–3.2] vs. 4.7[3.8–5.3], *P* < 0.001). Ultrasound measures confirmed body edema: skin-bone distance was increased in CLS patients (day 1 upper extremity: 0.7[0.5–1] vs. 0.4[0.3–0.5] cm, *P* < 0.001), echogenicity (day 1 upper extremity: 29[24–41] vs. 19[16–21], *P* < 0.001) and echo free space were higher (day 1 upper extremity: 0.2[0.1–0.4] vs. 0, *P* < 0.001), while more pulmonary B-lines (day 1: 4[3–5] vs. 2[1–3], *P* < 0.001) were counted. Ultrasound measures of edema showed strong correlation with results of body impedance electrical analysis (Spearman ρ 0.71, *P* < 0.001).

Endothelial biomarkers were increased in the serum of CLS patients: Angiopoietin-2 (day 1: 9.9[6–17] vs. 3.7[3–6] ng/mL, *P* < 0.001), VE-Cadherin (day 1: 2[1.5–2.7] vs. 1.7[1.4–2.1] ng/mL, *P* < 0.001), Syndecan-1 (day 1: 234[132–506] vs. 66[47–103] ng/mL, *P* < 0.001), Heparan sulfate (day 1: 4.3[3–6] vs. 3[2–4] ng/mL, *P* < 0.001), and ICAM-1 (day 1: 386[269–576] vs. 216[176–313] ng/mL, *P* < 0.001) were higher among CLS patients. HMGB-1 showed no statistical differences between the groups.

Serum–creatinine (day 1: 97[62–168] vs. 80[62–88] µmol/L, *P* < 0.001) and urea (day 1: 21[13–31] vs. 10[8–14] mmol/L, *P* < 0.001) were increased in the CLS group, and prothrombin time (day 1: 41[35–49] vs. 34[29–38] sec, *P* < 0.001) was prolonged.

Inflammatory biomarkers showed increased cytokine levels in CLS patients: TNF-α (day 1: 1.8[0–3] vs. 0.5[0–1] ng/L, *P* = 0.038), IL-1ß (day 1: 0.4[0–1.2] vs. 0.2[0–0.5] ng/L, *P* = 0.019), IL-6 (day 1: 164[66–385] vs. 90[40–212] ng/L, *P* = 0.001), IL-8 (day 1: 115[68–272] vs. 54[30–106] ng/L, *P* < 0.001), and IL-10 (day 1: 7[3–16] vs. 4[2–7] ng/L, *P* < 0.001) showed higher serum concentrations in CLS patients. No statistically significant differences in IL-12 were seen. White blood cell count was higher in CLS patients (day 1: 14[9–18] vs. 11[8–14] 10^3^/*µ*L, *P* = 0.001).

**Fig. 3:**
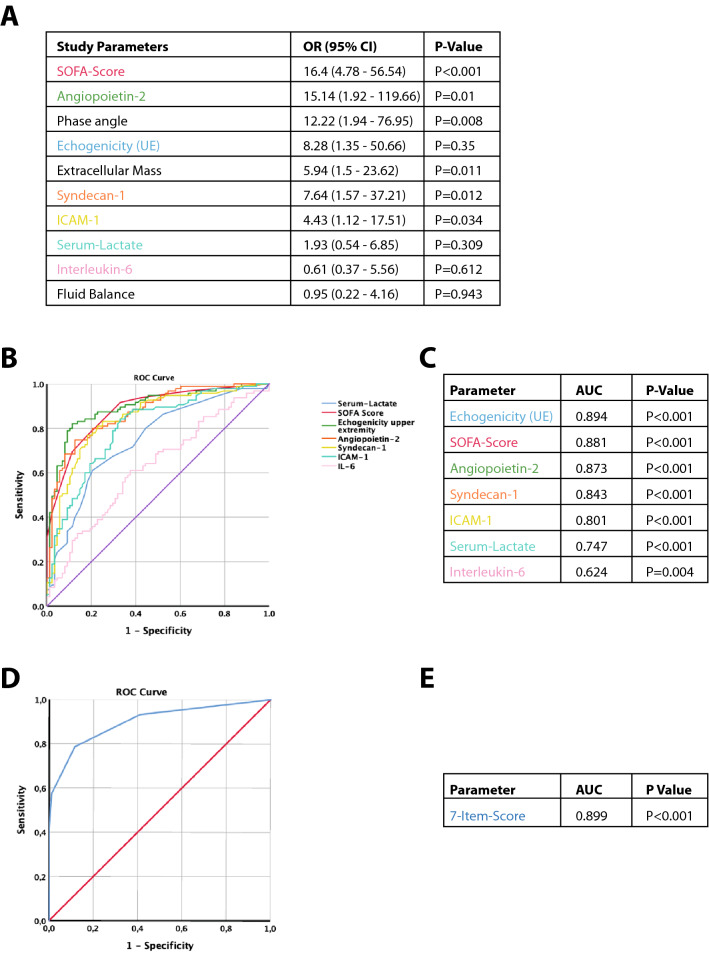
**A** Multivariate binary logistic regression for independent risk factor analysis.** B**,** C**. Evaluation of parameters from the multivariate analysis using Receiver Operating Characteristics (patient data from ICU day 1).** D**,** E**. Development of a 7-item scoring system (values > 75th percentile of cohort led to one point in score; *UE*  upper extremity)

Multivariate binary logistic regression showed significant results for the parameters SOFA-score (OR 16.4 [5–57], *P* < 0.001), angiopoietin-2 (OR 15.1 [2–120], *P* = 0.01), phase angle (OR 12.2 [2–77], *P* = 0.008), echogenicity of the upper extremity (OR 8.3 [1–51], *P* = 0.035), extracellular mass (OR 5.9 [2–24], *P* = 0.011), syndecan-1 (OR 7.6[2–37], *P* = 0.012), and ICAM-1 (OR 4.4 [1–18], *P* = 0.008; see Fig. 3A).

From multivariate analysis, parameters were evaluated regarding its discriminatory power for differentiating CLS and No-CLS patients (see Fig. 3B–E). As a single biomarker angiopoietin-2 with a set cutoff of 5.5 ng/mL showed an area under the ROC curve (AUC) of 0.873 (*P* < 0.001) leading to a diagnostic sensitivity of 0.796, a specificity of 0.747, a positive-predictive value (PPV) of 0.774 and a negative-predictive value (NPV) of 0.772.

A score was built using the seven parameters echogenicity, SOFA-Score, angiopoietin-2, syndecan-1, ICAM-1, lactate and IL-6 (with 1 point given for values above the 75^th^ percentile of the whole patient cohort). With a score cutoff of ≥ 2 (out of 7 possible points), the following characteristics are reported: AUC was 0.899 (*P* < 0.001) leading to a diagnostic sensitivity of 0.786, specificity of 0.884, PPV of 0.88 and NPV of 0.792.

Developing the scoring system further, a machine-learning based model was created to increase discriminatory power. In a first approach, two data sets including a variation of parameters were analyzed (see Additional File 6). This showed an AUC of 0.964 (*P* < 0.001) for 27-parameter approach in the random forest prediction model, while a serology-only approach led to an AUC of 0.882 (*P* < 0.001) in the same model. Using routinely available biological markers (i.e., parameters from the patients’ complete blood count, serum chemistry, etc.), an AUC of 0.685 (*P* = 0.002) was calculated.

**Fig. 4:**
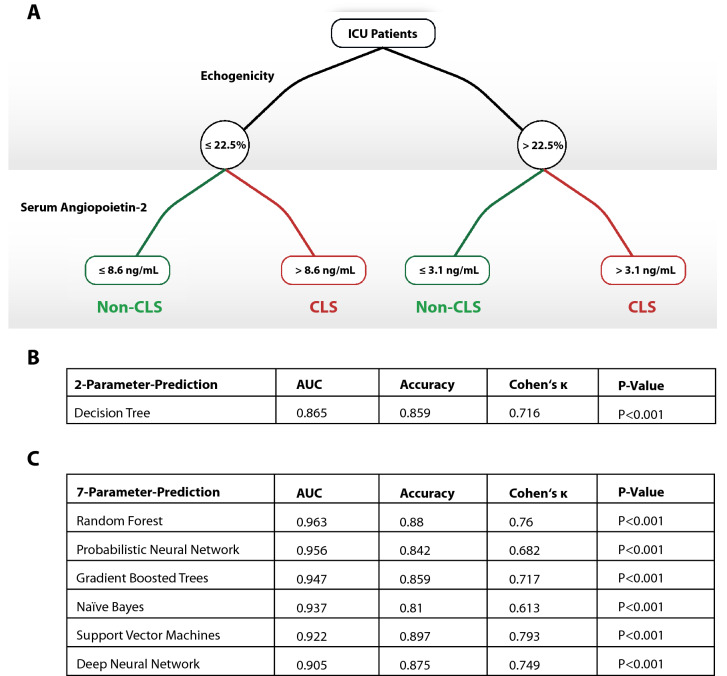
Machine learning based prediction model based on ICU data from day 1. **A**, **B** show a decision tree model which was computed from the 7-parameter input variables showing a reliable differentiation using only the two parameters of echogenicity and angiopoietin-2 level. **C** shows the comparison of diagnostic specifications of different machine-learning models computing the 7-parameter approach (echogenicity, SOFA-Score, angiopoietin-2, syndecan-1, ICAM-1, lactate, IL-6)

To increase clinical utility, the seven previously identified parameters, which are easily and non-invasively accessible were included into a new model (echogenicity, SOFA-Score, angiopoietin-2, syndecan-1, ICAM-1, lactate, IL-6; see Fig. 4A, B). First, a decision tree analysis was performed, showing good predictive characteristics with only two parameters; these are echogenicity and angiopoietin-2 level. Its AUC was 0.865 (*P* < 0.001), showing a sensitivity of 0.815, a specificity of 0.824, a PPV of 0.846 and a NPV of 0.798.

To further assess the diagnostic power, six different machine-learning algorithms (Random Forest, Gradient Boosted Trees, Probabilistic Neural Network, Naïves Bayes, Support Vector Machines, and Deep Neural Networks) were evaluated regarding its predictive capabilities for the promising 7-parameter data set (see Fig. 4C). This evaluation used tenfold cross validations to enable the contextualization of model performance on data that was not used for model fitting. The Random Forest Model showed the best discriminatory characteristics with an AUC of 0.963 (*P* < 0.001), an accuracy of 0.88, and a Cohen’s κ of 0.76. Its diagnostic characteristics were flagged with a sensitivity of 0.893, a specificity of 0.875, a PPV of 0.885, and a NPV of 0.884—thus showing the best characteristics.

## Discussion

The results from our study can be summarized as follows: (A) The clinical evaluation and discrimination of patients into CLS and No-CLS was supported by laboratory and technical findings in our cohort. (B) Using multivariate and ROC analyses of our measurements, various parameters showed significant results and were, therefore, included in a novel score (“*CLS-Score*”). (C) This score demonstrated high predictive value showing sufficient sensitivity and specificity. Machine-learning further enhanced its diagnostic power. (D) CLS showed characteristic properties in critically ill patients—being associated with an increased 30-day mortality compared to No-CLS patients.

By stating that “*… microvascular leak is not a mere byproduct of sepsis, but instead a major contributor to its morbidity and mortality*” [22], Goldenberg et al. shifted the focus of attention to the consequences of CLS for critically ill patients. Despite their conclusion that CLS is responsible for outcome, no sound clinical evidence for this association has been demonstrated so far. However, the underlying mechanism of CLS (i.e. inflammation, positive fluid balance etc.) have been negatively correlated with outcome among critically ill patients [23, 24, 30]. CLS likely reflects the pathologic state in the continuum of extracellular fluid exchange between the extravascular and intravascular compartments. In health, this exchange is essential, while its dysregulation may lead to the clinical picture of CLS [31]. Impeding any clinical investigation of this context, no definition of CLS has been established yet. Marx et al. previously characterized CLS as a loss of intravascular fluids to ‘third spaces’, thereby increasing edema and hemodynamic instability with the need for intravascular fluid replacement [1], while Cordemans et al. evaluated the Capillary Leak Index using the serum markers C-reactive protein and albumin [6]. In their attempt to diagnose CLS, Marx investigated six patients in septic shock by techniques, such as indocyanine green measurements, chromium-51 labeled erythrocytes, and colloid osmotic pressure. They concluded that measurement of extracellular water and the response to colloid osmotic pressure may help to identify CLS patients. Again, these findings have not led to any accepted definition. To facilitate a sustainable classification, various parameters involved in CLS’ pathophysiology and measurements which are easy-to-access were identified as an integral part of our scoring approach. To interpret our results’ prognostic relevance, we suggest to consider the CLS as an independent entity; not just a transient epiphenomenon in intensive care units.

Recent experimental investigations have shed light into the pathophysiological process of altered endothelial permeability in critical illness. Increased permeability may pre-dispose to organ dysfunction impairing capillary exchange process [4]. In general, edema (i.e. interstitial hypervolemia) from CLS is formed by fluid shift from intra- to extravascular space. Extravascular fluid returns back to the circulation by lymphatic pathways which can be influenced by drugs used in intensive care medicine [32]. With an approximated surface area of 7,000m^2^ [33], the endothelium accounts for a considerable area of exchange. Especially the cleavage of inter-endothelial adhesion (i.e. loss of cellular junction proteins) and shedding of glycocalyx have been described as milestones leading to CLS [4, 14]. By acknowledging the revised Starling principle, the integrity of the endothelial glycocalyx presents a major determinant of intravascular fluid homeostasis [34]. Flemming et al. showed that the cleaved endothelial junction protein VE-Cadherin was elevated in the serum of seven sepsis patients [35]. This was confirmed in our study for CLS patients. Angiopoietin-1/2 dysbalance was associated with vascular leak, with high angiopoietin-2 levels increasing barrier permeability [36, 37]. In sepsis, angiopoietin-2 levels correlated with severity of organ failure in critically ill children, while non-survivors showed higher angiopoietin-2 concentration [38]. Angiopoietin-2 was, furthermore, evaluated as a biomarker for sepsis and its sequelae [39, 40]. Importantly, CLS patients in our study showed increased levels of angiopoietin-2. In further attempts to understand CLS pathophysiology, an increase in inflammatory phenotype was identified to compromise endothelial glycocalyx [41]. Martin et al. demonstrated that critically ill patients show a high concentration of cleaved glycocalyx molecules in serum depending on disease severity [42]. To further sustain its relevance, Chappell et al. showed significant shedding of glycocalyx in patients being exposed to ischemia and reperfusion injury [15]. From our data, we can report an increase of syndecan-1 and heparan sulfate in our cohort of CLS patients. These pathophysiological aspects led to our hypothesis that measurement of endothelial biomarkers can be useful for a clinical definition of CLS, and were, therefore, included in our scoring system. According to our data, angiopoietin-2, VE-cadherin, ICAM-1 and syndecan-1 were specifically shown to be of high prognostic relevance—both in multivariate and ROC analyses.

Scores may present a good tool for straightforward diagnostics. However, they may carry disadvantages as they tend to simplify coming to the cost of “individualized and compassionate care”[43]. For our approach, we aimed to facilitate straightforward patient classification of a complex phenotype. We first examined the clinical differences between the patients with CLS and No-CLS. As a single biomarker, angiopoietin-2 showed the best predictive characteristics for CLS with an AUC of 0.873. To account for CLS pathophysiology, we proposed several factors to be included into our scoring system: A marker for edema (i.e., echogenicity measurement by ultrasound), microvascular malperfusion (serum-lactate), underlying inflammation (IL-6), endothelial biomarkers (angiopoietin-2, ICAM-1, syndecan-1) and the disease severity (SOFA-Score) were, therefore, combined to create a novel score (the “*CLS-Score*”). These parameters were selected due to their properties: non-invasive nature, easy to implement in a hospital-based laboratory setting, and the lack of special knowledge and equipment needed for practical execution. To enhance the utility of our score we used the widely available ultrasound to quantify the grade of edema which correlated well with BIA measurements. The authors appreciate that the insight provided by BIA may reveal a more sophisticated picture of extravascular hypervolemia and the composition of fluid compartments [28]. Our 7-parameter scoring system was flagged with sufficient diagnostic characteristics resulting in an AUC of 0.899. In an effort to prioritize and contextualize these parameters for CLS patients, we employed machine learning (ML) to further refine our CLS score. In a simplified approach, we studied a decision tree model which revealed promising results working with two parameters only: first, the grade of edema was determined followed by angiopoietin-2 measurement. The specifications of the 2-parameter approach (AUC 0.865) showed similar results to the 7-parameter score. Such a simplified, rule-based approach can be easily applied by healthcare professionals and could potentially be integrated into clinical decision making without the need for any additional hardware. We sought to further boost predictive power using more complex ML algorithms. The best predictive value among all algorithms was obtained using a Random Forest approach showing excellent characteristics with an AUC of 0.963. A standard laboratory-only approach led to less favorable diagnostic strength, stressing the need for more specific test. Given that the evaluation of these ML algorithms was performed using cross-validation experiments, while the other statistical methods were evaluated on the complete data set, the better performance of ML might be even more substantial than the raw AUC numbers suggest. Characterized as a sub-group of artificial intelligence methodology, ML may carry great potential [44] and enables enhanced diagnostics for Intensive Care Medicine [45]. The models used for our study can easily be incorporated into a patient-data management system facilitating easy diagnostics (with further possibilities of cohort-specific calibration). Random forest models are also rapidly re-trained, so that an adaptive system might be conceived in the future that continuously includes additional data acquired in a specific healthcare setting for a specific cohort or, alternatively, aggregated from multiple ICUs to further improve general predictive power. In addition, a mobile app-based development can be outlined.

Certain limitations have to be discussed. First, due to the study design an arbitrary, but clinically relevant classification of CLS was necessary (as utilized in everyday clinical routine). Therefore, we used the accepted characteristics of CLS, i.e., edema, intravascular hypovolemia, positive fluid balance and hemodynamic instability [1], and used this as a surrogate at the discretion of physicians with more than 10 year experience in intensive care medicine. No set cutoff values were defined. The aim of our study was to find common characteristics for CLS which necessitated this binary clinical classification. We reached our goal to support subjective perception with data and ML derived classification to create a score enabling an objective diagnosis.

Second, some patients may have already experienced their individual peak of vascular leak, while others potentially were to experience aggravation of CLS. Therefore, a longitudinal study approach was used evaluating the dynamics, revealing decreasing severity of study parameters in most patients over time. This dynamic approach needs to be clarified more in detail using longitudinal studies.

Third, it has to be kept in mind that our scoring system is derived from one single cohort. Due to matters of recruiting, a significant number of patients underwent prior abdominal surgery. Identified differences between the CLS and No-CLS cohort regarding higher blood loss during surgery and use of ß-Receptor-Blockers deserve further attention. It seems inevitable to study more data sets from different cohorts (including non-surgical ICU patients) for further validation.

Fourth, the groups of CLS and No-CLS patients were not demographically balanced. This was imminent to the study approach in which we identified characteristics in a heterogenous group of CLS patients independent of etiology, age or demographic specifics. Further studies are warranted to study demographic and patient subgroups (e.g. sepsis patients).

## Conclusions

CLS presents with distinct characteristics. It may be classified using objective, non-invasive parameters in critically ill patients. A scoring system based on seven criteria which have shown to be robust in statistical testing demonstrated efficacy in identifying CLS patients. Its prognostic value was enhanced by machine learning and was supported by data on increased 30-day mortality. The *CLS-Score* can easily be integrated into an ICU’s patient-data management system or any other digital application. Alternatively, a simplified two-parameter approach is offered using a decision tree to enhance clinical utility. Further studies are needed to determine the sequelae of diagnosing Capillary Leak Syndrome.

## Supplementary Information

**Additional file 1**. Supplemental methods section; More details regarding the methodology of the study.

**Additional file 2: Figure S1**. 30-day mortality was increased in the CLS group as revealed by the Kaplan–Meier survival curve.

**Additional file 3: Table S1**. Cause of mortality in patients deceased while participating in our study (CMO = comfort measures only).

**Additional file 4: Table S2**. Characteristics of healthy volunteers.

**Additional file 5: Table S3**. Univariate analysis of differences between CLS and No-CLS patients (SAPS II = Simplified Acute Physiology Score, Sequential Organ Failure Assessment Score = SOFA, Acute Physiology and Chronic Health Evaluation Score II = APACHE II, Intercellular Adhesion Molecule-1 = ICAM-1, High Mobility Group Box-1 = HMGB-1, Tumor Necrosis Factor = TNF, IL = Interleukin ; * 5–95 percentile derived from healthy volunteers.

**Additional file 6: Figure S2**. (A) 27-parameter machine learning models to predict CLS. (B) Serology-only machine-learning prediction of CLS.

## Data Availability

The data sets used and/or analysed during the current study are available from the corresponding author on reasonable request.

## Abbreviations

AUC: Area under the curve
CLS: Capillary leak syndrome
ICU: Intensive Care Unit
IL: Interleukin
ML: Machine Learning
NPV: Negative predictive value
PPV: Positive predictive value
ROC: Receiver operating characteristics
SOFA: Sequential organ failure assessment
