# Supplementary material for: Diagnosing capillary leak in critically ill patients: development of an innovative scoring instrument for non-invasive detection
Source: Ann Intensive Care. 2021 Dec 15;11:175. doi: 10.1186/s13613-021-00965-8 (PMC8674404; doi:10.1186/s13613-021-00965-8)
Supplement: Supplementary file 1 — Additional file 1. Supplemental methods section; More details regarding the methodology of the study. [file 13613_2021_965_MOESM1_ESM.docx]

**Supplemental Methods:**

Patient Classification:

The independent assessors were not involved in patient care, and were blinded to patient information other than grade of edema, signs of hypovolemia, positive fluid balance and hemodynamic instability. The assessment was made on the first day of the patient course on the ICU, and the assessors were not aware of the patient outcomes. If there was no agreement on patient classification by the two assessors, a third assessor was asked to evaluate the respective patient.

Data collection:

Daily measurements were performed in the ICU and data was archived in a Microsoft Access database (V2013, Washington, USA). Routinely available parameters like patients’ medication, reports, laboratory results and blood gas analyses were collected from the hospital-based patient data management systems (PDMS). Patients were studied daily with bioelectrical impedance analysis (BIA) and ultrasound measurements. Serum was collected daily and stored at -80°C until analysis. Patients were followed throughout the hospital stay and 30-day mortality was observed.

Ultrasound was performed using a GE Vivid S5 with a linear probe (GE Healthcare, Chicago, USA). Patients were examined in a standardized approach and images were acquired with the ultrasound pre-set for soft tissue examinations. For the upper extremity a standard image was acquired with a longitudinal image of the 2^nd^ metacarpal bone from the dorsal side of the hand. Next, a longitudinal image of the 2^nd^ rib was acquired. The probe was moved to acquire an image of the lung to count pulmonary B-lines. For the lower extremity, the 1^st^ metatarsal bone was analysed. In an offline analysis respective skin-to-bone distance was measured. Images were stored to calculate echogenicity using ImageJ, measuring the mean brightness of the subcutaneous tissue (ImageJ, NIH, USA).

Analyses of serum included enzyme-linked immunosorbent assay (ELISA) and fluorescence-activated cell sorting (FACS). ELISA kits were used to determine serum concentrations according to the manufacturer’s recommendation (Angiopoietin-2 [Human Angiopoietin-2 Quantikine ELISA kit, #DANG20, R&D Systems, USA]; VE-Cadherin [Human VE-Cadherin Quantikine ELISA kit, #DCADV0, R&D Systems, USA]; heparan sulfate [Heparan Sulfate Assay Kit, #SEA565Hu, Cloud-Clone Corp., USA]; syndecan1 [Human Syndecan-1 ELISA kit, #AB46506, Abcam Ltd, United Kingdom]; ICAM-1 [Human ICAM-1 Quantikine ELISA kit, #DCD540, R&D Systems, USA]; HMGB-1 [Human HMGB-1 ELISA, #ST51011, Tecan Trading AG, Switzerland]). For analysis of TNF-α, IL-1ß, IL-6, IL-8, IL-10 and IL-12p70, a bead array kit was used for simultaneous analysis of cytokines from one serum sample using FACS (Human Inflammatory Cytokine Kit, #551811, BD^TM^, USA).

Statistical Analysis:

The sample size for our study was preemptively calculated in collaboration with the Institute for Medical Biometry and Statistics at the Medical Center – University of Freiburg (Prof. Dr. D. Hauschke, hauschke@imbi.uni-freiburg.de). Assuming an effect size of 0.4 based on preliminary data, a power of at least 80% and an alpha of 5%, a sample size of 100 patients per group was determined in order to detect a statistically significant difference.
