# Supplementary material for: Diagnosing capillary leak in critically ill patients: development of an innovative scoring instrument for non-invasive detection
Source: Ann Intensive Care. 2021 Dec 15;11:175. doi: 10.1186/s13613-021-00965-8 (PMC8674404; doi:10.1186/s13613-021-00965-8)
Supplement: Supplementary file 3 — Additional file 3: Table S1. Cause of mortality in patients deceased while participating in our study (CMO = comfort measures only). [file 13613_2021_965_MOESM3_ESM.docx]

**Supplementary Table 1:**

| **No.** | **Group** | **Cause of death** | **Comment** |
| --- | --- | --- | --- |
| 1 | No-CLS | Anastomotic dehiscence after hemicolectomy | Patient’s decision against re-laparotomy, CMO |
| 2 | CLS | Septic shock due to pneumonia |  |
| 3 | CLS | Septic shock due to pneumonia | Status post extensive surgery and intraperitoneal chemotherapy |
| 4 | CLS | ARDS |  |
| 5 | CLS | Septic shock due to renal tract infection | Apparent refractory hemodynamic instability |
| 6 | CLS | Septic shock due to intraperitoneal infection | Status post hemicolectomy |
| 7 | CLS | Septic shock due to pneumonia |  |
| 8 | CLS | Acute-on-chronic heart failure | Death despite immediate cardiopulmonary resuscitation |
| 9 | CLS | ARDS |  |
| 10 | CLS | Septic shock due to intraperitoneal infection | Progression of cholangiocarcinoma, CMO |
| 11 | CLS | ARDS |  |
| 12 | CLS | Septic shock of unclear etiology |  |
| 13 | CLS | Septic shock due to intraperitoneal infection | Status post hemi-hepatectomy, advanced hepatocellular carcinoma |

**Suppl. Table 1:** Cause of mortality in patients deceased while participating in our study (CMO = comfort measures only)
