# Supplementary material for: Diagnosing capillary leak in critically ill patients: development of an innovative scoring instrument for non-invasive detection
Source: Ann Intensive Care. 2021 Dec 15;11:175. doi: 10.1186/s13613-021-00965-8 (PMC8674404; doi:10.1186/s13613-021-00965-8)
Supplement: Supplementary file 4 — Additional file 4: Table S2. Characteristics of healthy volunteers. [file 13613_2021_965_MOESM4_ESM.docx]

**Supplementary Table 2:**

| **Parameter** | **Volunteers (N=30)** |
| --- | --- |
| Male (%) | 15 (50 %) |
| Age (median±IQR) | 22 (22-24) |
| Weight, kg (median±IQR) | 68 (61-73) |
| Height, cm (median±IQR) | 175 (171-180) |
| BMI, (median±IQR) | 22 (20-23) |

**Suppl. Table 2:** Characteristics of healthy volunteers
