# Supplementary material for: Diagnosing capillary leak in critically ill patients: development of an innovative scoring instrument for non-invasive detection
Source: Ann Intensive Care. 2021 Dec 15;11:175. doi: 10.1186/s13613-021-00965-8 (PMC8674404; doi:10.1186/s13613-021-00965-8)
Supplement: Supplementary file 5 — Additional file 5: Table S3. Univariate analysis of differences between CLS and No-CLS patients (SAPS II = Simplified Acute Physiology Score, Sequential Organ Failure Assessment Score = SOFA, Acute Physiology and Chronic Health Evaluation Score II = APACHE II, Intercellular Adhesion Molecule-1 = ICAM-1, High Mobility Group Box-1 = HMGB-1, Tumor Necrosis Factor = TNF, IL = Interleukin ; * 5–95 percentile derived from healthy volunteers. [file 13613_2021_965_MOESM5_ESM.docx]

**Supplementary Table 3:**

|  | Reference range (volunteers, 5 – 95 percentile) | Day on ICU | No-CLS (N=95) | CLS (N=100) | *P* Value |
| --- | --- | --- | --- | --- | --- |
| ***Hemodynamics and Fluids:*** | | | | | |
| Catecholamines (%) |  | 1 | 81 (85 %) | 95 (95 %) | **0.022** |
|  |  | 2 | 16 (17 %) | 86 (86 %) | **<0.001** |
| Norepinephrine (>18µg/min), (%) |  | 1 | 1 (1 %) | 58 (58 %) | **<0.001** |
|  |  | 2 | 0 | 58 (58 %) | **<0.001** |
| Dobutamine, (%) |  | 1 | 1 (1 %) | 13 (13 %) | **0.001** |
|  |  | 2 | 0 | 14 (14 %) | **<0.001** |
| Epinephrine, (%) |  | 1 | 0 | 4 (4 %) | 0.123 |
|  |  | 2 | 0 | 5 (5 %) | **0.031** |
| Vasopressin, (%) |  | 1 | 0 | 5 (5 %) | 0.061 |
|  |  | 2 | 0 | 4 (4 %) | 0.054 |
| Fluid balance, mL (median±IQR) |  | 1 | 812 (140-1437) | 1613 (345-3153) | **<0.001** |
|  |  | 2 | 445 (70-980) | 1267 (222-2613) | **0.001** |
| Fluid input, mL  median±IQR) |  | 1 | 2077 (1421-2945) | 3280 (2226-4854) | **<0.001** |
|  |  | 2 | 2049 (1575-2694) | 2967 (2024-3975) | **<0.001** |
| Urine output, mL (median±IQR) |  | 1 | 1250 (775-1825) | 1400 (861-2485) | **0.034** |
|  |  | 2 | 1576 (900-2195) | 1500 (815-2590) | 0.234 |
| Lactate, mmol/L (median±IQR) | <1.5 | 1 | 0.9 (0.7-1.2) | 1.5 (1-2.2) | **<0.001** |
|  |  | 2 | 0.8 (0.6-1.1) | 1.3 (0.8-2) | **<0.001** |
| Hemoglobin, g/dL (median±IQR) | 12 – 17 | 1 | 10.6 (8.5-12.1) | 8.6 (8-9.8) | **<0.001** |
|  |  | 2 | 9.9 (8.4-11.5) | 8.3 (8-9.5) | **<0.001** |
| ***Scoring:*** | | | | | |
| SAPS II (median±IQR) |  | 1 | 19 (13-24) | 36 (28-45) | **<0.001** |
|  |  | 2 | 18 (13-24) | 35 (27-43) | **<0.001** |
| SOFA (median±IQR) |  | 1 | 1 (0-3) | 6 (4-9) | **<0.001** |
|  |  | 2 | 1 (0-3) | 6 (4-8) | **<0.001** |
| APACHE II (median±IQR) |  | 1 | 6 (4-9) | 12 (9-16) | **<0.001** |
|  |  | 2 | 6 (4-8) | 12 (8-16) | **<0.001** |
| ***Body impedance electrical analysis:*** | | | | | |
| Metabolic rate at rest, kcal (median±IQR) | (1506 – 1656)* | 1 | 1505 (1360-1670) | 1260 (1050-1390) | **<0.001** |
|  |  | 2 | 1510 (1360-1650) | 1310 (1070-1450) | **<0.001** |
| Phase Angle (median±IQR) | (6.2 – 6.8)* | 1 | 4.7 (3.8-5.3) | 2.6 (2-3.2) | **<0.001** |
|  |  | 2 | 4.7 (3.9-5.4) | 2.8 (2-3.2) | **<0.001** |
| Body water, L (median±IQR) | (38 – 44)* | 1 | 47 (41-52) | 51 (45-59) | **0.001** |
|  |  | 2 | 46 (40-51) | 52 (45-61) | **<0.001** |
| Net weight, kg (median±IQR) | (52 – 60)* | 1 | 64 (56-70) | 70 (61-83) | **<0.001** |
|  |  | 2 | 63 (56-70) | 71 (62-81) | **<0.001** |
| Extracellular Mass, kg (median±IQR) | (24 – 27)* | 1 | 35 (30-38) | 50 (43-63) | **<0.001** |
|  |  | 2 | 35 (30-38) | 50 (43-63) | **<0.001** |
| Extracellular Water, L (median±IQR) | (15 – 18)* | 1 | 21 (18-24) | 26 (22-32) | **<0.001** |
|  |  | 2 | 21 (18-24) | 26 (24-32) | **<0.001** |
| Body Cellular Mass, L (median±IQR) | (28 – 33)* | 1 | 28 (23-33) | 19 (14-25) | **<0.001** |
|  |  | 2 | 28 (38-49) | 22 (13-26) | **<0.001** |
| Fraction of Cells, % (median±IQR) | (53 – 55)* | 1 | 44 (38-49) | 30 (21-34) | **<0.001** |
|  |  | 2 | 45 (38-49) | 31 (21-35) | **<0.001** |
| Body fat, kg (median±IQR) | (10 – 13)* | 1 | 10 (5-18) | 6 (1-13) | **0.001** |
|  |  | 2 | 10 (6-18) | 5 (1-15) | **0.001** |
| ***Ultrasound:*** | | | | | |
| Skin-Bone distance upper extremity, cm (median±IQR) | (0.21 – 0.23)* | 1 | 0.36 (0.3-0.5) | 0.65 (0.5-1) | **<0.001** |
|  |  | 2 | 0.35 (0.3-0.4) | 0.65 (0.5-1) | **<0.001** |
| Skin-Bone distance lower extremity, cm (median±IQR) | (0.31 – 0.35)* | 1 | 0.42 (0.3-0.5) | 0.59 (0.5-0.8) | **<0.001** |
|  |  | 2 | 0.4 (0.3-0.5) | 0.58 (0.4-0.8) | **<0.001** |
| Skin-Bone distance Rib, cm (median±IQR) | (1.2 – 1.3)* | 1 | 1.2 (1-1.5) | 1.3 (1.1-1.5) | 0.139 |
|  |  | 2 | 1.2 (0.9-1.5) | 1.3 (1.1-1.6) | **0.048** |
| Echogenicity upper extremity (median±IQR) | (18 – 20)* | 1 | 19 (16-21) | 29 (24-41) | **<0.001** |
|  |  | 2 | 18 (16-21) | 27 (22-38) | **<0.001** |
| Echogenicity lower extremity (median±IQR) | (19 – 21)* | 1 | 19 (16-22) | 28 (24-33) | **<0.001** |
|  |  | 2 | 19 (16-22) | 28 (23-33) | **<0.001** |
| Echogenicity rib (median±IQR) | (22 – 24)* | 1 | 26 (24-29) | 33 (29-37) | **<0.001** |
|  |  | 2 | 25 (23-27) | 32 29-36) | **<0.001** |
| Echo free space upper extremities (median±IQR) | 0* | 1 | 0 | 0.15 (0.1-0.4) | **<0.001** |
|  |  | 2 | 0 | 0.1 (0-0.3) | **<0.001** |
| Echo free space lower extremities (median±IQR) | 0* | 1 | 0 | 0.1 (0-0.2) | **<0.001** |
|  |  | 2 | 0 | 0.05 (0-0.2) | **<0.001** |
| Echo free space rib (median±IQR) | 0* | 1 | 0 | 0.1 (0-0.2) | **<0.001** |
|  |  | 2 | 0 | 0.1 (0-0.1) | **<0.001** |
| Pulmonary B-lines (median±IQR) | (2 – 3)* | 1 | 2 (1-3) | 4 (3-5) | **<0.001** |
|  |  | 2 | 2 (1-3) | 4 (2-5) | **<0.001** |
| ***Laboratory analyses:*** | | | | | |
| Angiopoietin-2, ng/mL (median±IQR) | (2.21 – 3)* | 1 | 3.71 (2.6-5.6) | 9.94 (6.2-17.3) | **<0.001** |
|  |  | 2 | 3.64 (2.8-5.1) | 9.99 (6.8-16.4) | **<0.001** |
| VE-Cadherin, ng/mL (median±IQR) | (2.99 – 3.45)* | 1 | 1.65 (1.4-2.1) | 1.98 (1.5-2.7) | **<0.001** |
|  |  | 2 | 1.75 (1.4-2.2) | 1.98 (1.5-2.5) | **0.047** |
| Syndecan-1, ng/mL (median±IQR) | (47 – 99)* | 1 | 66 (47-103) | 234 (132-506) | **<0.001** |
|  |  | 2 | 68 (46-113) | 287 (124-535) | **<0.001** |
| Heparan sulfate, µg/mL (median±IQR) | (3.9 – 4.78)* | 1 | 2.97 (2.3-4) | 4.31 (3-6.1) | **<0.001** |
|  |  | 2 | 3.18 (2.5-4.3) | 4.66 (3.3-7.1) | **<0.001** |
| ICAM-1, ng/mL (median±IQR) | (165 – 193)* | 1 | 216 (176-313) | 386 (269-576) | **<0.001** |
|  |  | 2 | 224 (184-323) | 425 (288-634) | **<0.001** |
| HMGB-1, ng/mL (median±IQR) | (2.6 – 4.1)* | 1 | 12 (7-23) | 13 (8-23) | 0.758 |
|  |  | 2 | 14 (9-20) | 12 (8-19) | 0.256 |
| Urea, mmol/L (median±IQR) | 5 – 15 | 1 | 10 (8-14) | 21 (13-31) | **<0.001** |
|  |  | 2 | 10 (8-14) | 21 (14-36) | **<0.001** |
| Creatinine, µmol/L (median±IQR) | 45 – 103 | 1 | 80 (62-88) | 97 (62-168) | **<0.001** |
|  |  | 2 | 80 (62-88) | 97 (62-168) | **0.003** |
| INR (median±IQR) | 0.85 – 1.15 | 1 | 1.1 (1-1.1) | 1.1 (1-1.2) | **0.001** |
|  |  | 2 | 1 (1-1.1) | 1.1 (1-1.2) | **0.002** |
| PTT, sec (median±IQR) | 25.1 – 37.7 | 1 | 34 (29-38) | 41 (35-49) | **<0.001** |
|  |  | 2 | 35 (31-37) | 43 (36-53) | **<0.001** |
| Platlet count, 10^3^/µl (median±IQR) | 150 – 400 | 1 | 201 (156-270) | 187 (106-318) | 0.316 |
|  |  | 2 | 180 (148-237) | 196 (117-284) | 0.657 |
| ***Inflammation:*** | | | | | |
| TNF-α, ng/L (median±IQR) | (0.18 – 0.66)* | 1 | 0.52 (0-1) | 1.8 (0-3.3) | **0.038** |
|  |  | 2 | 0.53 (0-0.7) | 0.82 (0.2-1.4) | 0.196 |
| IL-1ß, ng/L (median±IQR) | (0.04 – 0.27)* | 1 | 0.16 (0-0.5) | 0.37 (0-1.2) | **0.019** |
|  |  | 2 | 0.46 (0-1.6) | 0.66 (0-2.6) | **0.011** |
| IL-6, ng/L (median±IQR) | (5 – 11)* | 1 | 90 (40-212) | 164 (66-385) | **0.001** |
|  |  | 2 | 56 (23-105) | 120 (58-234) | **<0.001** |
| IL-8, ng/L (median±IQR) | (11 – 19)* | 1 | 54 (30-106) | 115 (68-272) | **<0.001** |
|  |  | 2 | 49 (35-95) | 141 (87-281) | **<0.001** |
| IL-10, ng/L (median±IQR) | (0.16 – 0.5)* | 1 | 4 (2-7) | 7 (3-16) | **<0.001** |
|  |  | 2 | 2.5 (1.1-4.8) | 5.5 (3.1-9.8) | **<0.001** |
| IL-12, ng/L (median±IQR) | (0.17 – 0.67)* | 1 | 0.42 (0-2) | 0.71 (0-1.1) | 0.055 |
|  |  | 2 | 0.57 (0-2.7) | 0.63 (0-2) | 0.294 |
| White blood cell count, 10^3^/µL (median±IQR) | 4 – 10 | 1 | 11 (8-14) | 14 (9-18) | **0.001** |
|  |  | 2 | 10 (8-14) | 14 (9-18) | **<0.001** |

**Suppl. Table 3:** Univariate analysis of differences between CLS and No-CLS patients (SAPS II = Simplified Acute Physiology Score, Sequential Organ Failure Assessment Score = SOFA, Acute Physiology and Chronic Health Evaluation Score II = APACHE II, Intercellular Adhesion Molecule-1 = ICAM-1, High Mobility Group Box-1 = HMGB-1, Tumor Necrosis Factor = TNF, IL = Interleukin ; * 5-95 percentile derived from healthy volunteers
